# Non-Invasive Mapping of Ventricular Action Potential Reconstructed from Contactless Magnetocardiographic Recordings in Intact and Conscious Guinea Pigs

**DOI:** 10.3390/jcdd12090343

**Published:** 2025-09-06

**Authors:** Riccardo Fenici, Marco Picerni, Peter Fenici, Donatella Brisinda

**Affiliations:** 1 School of Medicine and Surgery, Catholic University of the Sacred Heart, 00168 Rome, Italy; peter.fenici@unicatt.it; 2 Biomagnetism and Clinical Physiology International Center, 00144 Rome, Italy; mpicerni@sissa.it; 3 Mathematics Area, SISSA—International School for Advanced Studies, 34136 Trieste, Italy; 4 General Medicine and Aging Unit, Fondazione Policlinico Universitario A. Gemelli, Istituto di Ricerca e Cura a Carattere Scientifico (IRCCS), 00168 Rome, Italy

**Keywords:** magnetocardiography, non-invasive ventricular action potential reconstruction from magnetic signals, monophasic action potential, multimodal imaging of electrophysiologic events, guinea pig, magnetoionography

## Abstract

Optical mapping, nanotechnology-based multielectrode arrays and automated patch-clamp allow transmembrane voltage mapping with high spatial resolution, as well as L-type calcium and inward rectifier currents measurements using native mammalian cardiomyocytes. However, these methods are limited to in vitro and ex vivo experiments, while magnetocardiography (MCG) might offer a novel approach for non-invasive preclinical safety assessments of new drugs in intact and even conscious rodents by reconstructing the ventricular action potential waveform (rVAPw) from MCG signals. **Objective:** This study aims to assess the feasibility of rVAPw reconstruction from MCG signals in Guinea pigs (GPs) and validate the results by comparison with simultaneously recorded epicardial ventricular monophasic action potentials (eVMAP). **Methods:** Unshielded MCG (uMCG) data of 18 GPs, investigated anaesthetized and awake at ages of 5, 14, and 26 months using a 36-channel DC-SQUID system, were analyzed to calculate rVAPw from MCG’s current arrow map. **Results:** Successful rVAPw reconstruction from averaged MCG showed good alignment with eVMAP waveforms. However, some rVAPw displayed incomplete or distorted repolarization at sites with lower MCG amplitude. **Conclusions:** 300-s uMCG averaging allowed rVAPw reconstruction in intact GPs. Occasionally distorted rVAPw suggests the need for dedicated MCG devices development, with higher density of optimized vector sensors, and modelling tailored for small animal hearts.

## 1. Introduction

Since the 1950s, cardiac action potential (AP) recording in animal models has been widely used in experimental cardiac electrophysiology and preclinical safety assessments of new drugs [1]. Later, optical mapping techniques enabled the simultaneous recording of multiple APs with higher spatial resolution, facilitating the detection of regional repolarization heterogeneity, conduction dynamics, and calcium transient behaviour [2,3]. In recent years, advancements in nanotechnology-based multielectrode arrays have significantly improved the transmembrane voltage mapping of excitable cells. Additionally, automated patch-clamp technologies now allow for the recording of action potentials, L-type calcium currents, and basal inward rectifier currents in native mammalian cardiomyocytes for drug screening [4]. However, these methods are limited to in vitro or ex vivo experiments.

In the search for efficient methods to bridge the gap between experimental and clinical electrophysiology, catheter techniques for clinical recording of monophasic action potentials (MAP) were introduced in the 1970s [5,6,7] and have been extensively studied since [8,9,10,11,12,13]. Despite its documented utility [14], MAP recording is invasive and provides only a limited number of simultaneous recording points [15], thus offering limited spatial resolution compared to other techniques used in experimental animal studies. In contrast, contactless mapping of the cardiac magnetic field, known as magnetocardiography (MCG), has shown promise in both experimental animal models [16,17,18,19,20] and human patients [21,22]. Moreover, the seminal work of Kandori et al. suggests that MCG holds significant potential to enhance non-invasive electrophysiological assessment by mapping the magnetically reconstructed ventricular action potential waveform (rVAPw) [23].

However, the published reconstruction of the transmembrane action potential waveform from MCG is limited to a few reported human cases [23,24] and, to the best of our knowledge, has not yet been attempted in live, intact experimental animals. This study aims to evaluate the feasibility and limitations of reconstructing the ventricular action potential waveform from MCG in Guinea pigs (GPs) as an initial step toward enhancing the potential of contactless MCG mapping to become a reliable, non-invasive method for in vivo mapping of action potentials with accuracy comparable to experimental optimal mapping techniques without animal sacrifice.

The unique availability of simultaneous MCG mapping and epicardial ventricular monophasic action potential (eVMAP) recording in our experimental MCG database [25] allowed for preliminary validation of the magnetic rVAPw by direct comparison with the eVMAP waveform. Subsequently, the same computational procedure was applied to assess the variability of the magnetic reconstruction of ventricular action potential waveforms related to the age, gender, and weight of the rodents, both under anaesthesia and when awake.

## 2. Materials and Methods

The study is a retrospective analysis of MCG signals data available in our MCG database with a custom mathematical processing based on the method originally proposed by Kandori et al. [23], integrated with the multimodal electrophysiological imaging developed in our biomagnetism research center, as described in the following subsections. The original experimental data acquisition had been approved by the institutional Ethical Committee and complies with the Guide for the Care and Use of Laboratory Animals and the ARRIVE guidelines [26,27].

### 2.1. Animals

MCG data from 18 GPs (8 males and 10 females), who underwent multiple unshielded MCG (uMCG) studies at mean ages of 5, 14, and 26 months (between 2004 and 2006), were selected for this study and retrospectively analyzed to reconstruct the ventricular action potential waveform [23].

### 2.2. Experimental Setup and Measurement Techniques

#### 2.2.1. Unshielded MCG Recording Protocol and Postprocessing

**Figure 1:**
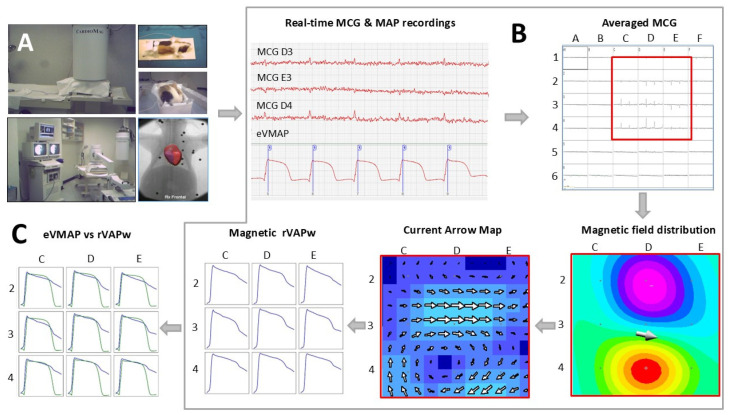
(**A**) Overview of the experimental setup; (**B**) Schematic of the data acquisition sequence, post-processing, and calculation for the non-invasive reconstruction of the ventricular action potential waveform (rVAPw) from MCG signals. The magnetic field distribution and the current arrow map refer to the 3 × 3 grid with the highest MCG signal (highlighted by the red square); (**C**) Overlay of averaged rVAPw (blue) and epicardial ventricular monophasic action potential (green) waveforms.

All animals were studied awake without sedation (in a prone position) and in separate sessions under anaesthesia with intramuscular ketamine (50 mg kg^−1^ body weight) and diazepam (2 mg kg^−1^ body weight). They were placed in a supine position on a small cradle and covered with surgical clothing to prevent any possible contact with the MCG instrumentation (Figure 1A).

MCG mapping was performed using a 36-channel system (CardioMag Imaging, Inc., Schenectady, NY, USA) at the Catholic University’s unshielded Biomagnetism Center, which is fully equipped for interventional electrophysiology (Figure 1A). The z-component of the cardiac magnetic field (Bz) was recorded with 36 direct-current superconducting quantum interference device (DC-SQUID) sensors, coupled to second-order axial gradiometers (featuring a 50 mm baseline and 20 mm pick-up coil’s diameter), enclosed in a cylindrical cryostat. The sensors were arranged in a square 20 × 20 cm array with a 4 cm pitch (centre-to-centre). The average system effective sensitivity was 50 fT/√Hz from 1 Hz within the frequency range of DC–500 Hz, depending on the sensors’ tuning accuracy and the level of environmental noise [28]. All signals were digitally recorded (24-bit conversion at a 1 kHz sampling frequency) in both the supine and prone positions.

The distance between the bottom of the cryostat and the animal’s chest was kept constant (15 mm) for all animals, in both supine and prone positions. All recordings were performed under normal sinus rhythm and spontaneous breathing. uMCG mapping typi cally lasted 300 s. The repeatability and reproducibility of MCG recordings were tested as described in [29]. At the end of the procedure under anaesthesia, the animals were monitored until they fully regained consciousness and were then returned to the animal lodge facility.

To optimize the MCG signal-to-noise ratio before the reconstruction of time-variant magnetic field dynamics during sinus rhythm, post-processing of the MCG signals included digital filtering (using a selective COMB filter to remove 50 Hz power line noise and a low-pass filter at 100 Hz) and time averaging, typically over 300 s. For magnetic reconstruction of the ventricular action potential waveform, the reference baseline was manually set before the QRS onset.

After calculating the magnetic field distribution (MFD), the current arrow map (CAM) was computed according to Hosaka Cohen [30] and was automatically visualized onto the recording grid in a plane parallel to the anterior chest wall.

Although the CAM provides a current pattern of the heart without the need to solve a nonlinear inverse problem, the inverse solution using the magnetic dipole model was also calculated for three-dimensional (3D) MCG localization of ventricular sources, as part of the 3D reconstruction of the animal’s heart model [31], derived from two orthogonal fluoroscopic images, which were acquired with markers corresponding to the MCG sensor positions (Figure 1A).

#### 2.2.2. Epicardial MAP Recording with the Amagnetic Catheter Technique

The patented multipurpose amagnetic catheter for MCG-compatible simultaneous multiple MAP (MultiMAP) recording [15,32] features a variable number of non-polarizable amagnetic electrodes at the tip, capable of simultaneously recording four monophasic action potentials and performing local cardiac pacing. The 3D position of the catheter’s tip was automatically detected and visualized using the Magnetic Source Imaging (MSI) method [33]. The accuracy of MSI for source localization was previously reported [34,35,36].

High-resolution MultiMAP recordings, with an inter-MAP electrode distance of 1.2 mm, were differentially amplified (bandwidth DC-500 Hz) and digitized at 1 kHz (CardioLab GE Medical System). All tip electrodes were connected to the positive input of high-impedance DC-coupled optically isolated differential preamplifiers, while the reference electrode was connected to the negative input of the amplifier. With this recording setup, MAP signals are oriented upward (Figure 1B).

In animals with simultaneous MCG and MAP recordings, one of the MAP analogue signals was connected to the MCG mapping system to synchronize the CardioMag with the Cardiolab data acquisition and to trigger MCG signal averaging [37] (Figure 1B). In the absence of MAP signals (i.e., when the animals were investigated only non-invasively with MCG), a single-lead ECG signal was used to trigger the MCG signal averaging, as usually performed during non-invasive MCG mapping.

#### 2.2.3. Reconstruction of the Ventricular Action Potential from uMCG Signals

To reconstruct the ventricular action potential waveform from averaged MCG data, the method described by Kandori et al. [23] was applied. This method is based on the relationship between the ionic currents (inward and outward) of the cell membrane, which occur during the depolarization and repolarization phases, respectively, and generate the waveforms of both the action potential and the ECG.

The electrical propagation of the cell membrane was calculated using the heart conduction system model by Beeler and Reuter [38], along with the assumption that the MCG waveform reflects the spatial electrical activation of the heart. Since both ECG and MCG signals are measured at a distance from the current flow, they are associated with the derivative waveforms of the action potential generated by the current flow.

The components *I_x_* and *I_y_* of the CAM at each site are derived by approximating the partial derivatives of the normal component of the magnetic field *B_z_* using the following expressions:

$$I_{x}=\frac{\partial B_{z}}{\partial y}$$

and

$$I_{y}=-\frac{\partial B_{z}}{\partial x}.$$

The magnitude of the current vectors, representing the intensity of the current, is calculated as the Euclidean norm:

$$\sqrt{{I_{x}}^{2}+{I_{y}}^{2}}.$$

In this way, the CAM provides a dynamic, time-resolved visualization of the current pattern in the heart, avoiding the need to solve a nonlinear inverse problem.

Then, assuming a uniform cell-to-cell gap junction resistance (set to 1), the ventricular action potential waveform can be reconstructed by summing and subtracting electrical currents during different phases of the cardiac cycle.

During phase 0 (depolarization), currents from the onset to the end of the QRS complex (ventricular depolarization) are summed, starting from an initial value of zero and continuing until the end of the QRS (AP maximum). This summation reflects the rapid influx of Na+ ions that initiates and propagates the action potential.

During phases 1 to 3 (repolarization), currents are subtracted starting from the AP maximum value at the end of depolarization (QRS_end_) to the end of the T wave (T_end_). This subtraction represents the outward flow of K^+^ ions during ventricular repolarization, gradually returning the membrane to its resting potential [23].

#### 2.2.4. Preliminary Validation of the Magnetically rVAPw by Comparison with Simultaneously Recorded eVMAP

The data from eight sessions with simultaneous MCG and ventricular MAP recordings were preliminarily analyzed to evaluate the correlation between action potential waveforms reconstructed from MCG mapping (rVAPw) and those obtained from epicardial ventricular MAP (eVMAP) recordings using the amagnetic catheter technique [10,15] (Figure 1C).

A custom software was developed to analyze the rVAPw and eVMAP signals by automatically detecting the baseline, upstroke, peak, and offset of both signals. It also calculated the duration of phase 0, as well as the rVAPw and eVMAP durations measured at 30% (d30%), 50% (d50%), and 80% (d80%) repolarization levels. APD80 [3] was chosen to also allow measurements of rVAPw duration in few cases of incomplete repolarization or unstable baseline. These measurements are independent of scaling factors and the selected baseline value, ensuring they remain unaffected by the specific choice of the resistance factor (r = 1), as long as the uniformity assumption for r holds. Given the fixed position of the MAP electrodes, the direction of the local depolarization wavefront is also detectable [25].

#### 2.2.5. Statistical Analysis

Statistical analysis was performed using SPSS (Version 21.0). Continuous variables are presented as mean ± standard deviation. Differences between groups were assessed using Student’s *t*-test (paired or unpaired, as appropriate) and one-way ANOVA to compare the results across the three groups. A *p*-value of <0.05 was considered statistically significant.

## 3. Results

### 3.1. uMCG Postprocessing and Ventricular Action Potential Waveform Reconstruction

At the age of 12 months, the weight was 643 ± 23.9 g (males), 570 ± 12.4 g (females). The average cardiac magnetic field intensity (picotesla) at the QRS peak was 5.04 ± 2.56 (males) and 4.18 ± 2.73 (females). After adaptive digital filtering and 300 s of signal averaging, the signal-to-noise (S/N) ratio of the uMCG signals within the DC-100 Hz bandwidth was sufficient for reproducible magnetic field analysis, calculation of the MFD, CAM, and magnetic reconstruction of the ventricular action potential in all animals selected for this study.

### 3.2. Preliminary Validation of the Magnetically rVAPw by Comparison with Simultaneously Recorded eVMAP

Data from eight recording sessions with high-quality simultaneous MCG mapping and eVMAP recordings were available. The comparative evaluation of Phase 0, d30%, d50%, and d80% of rVAPw and eVMAP, calculated after normalizing the rVAPw amplitude to that of eVMAP, is summarized in Table 1.

The table highlights the general agreement but also shows quantitative differences in the duration of both depolarization and repolarization parameters between eVMAP and rVAPw. In some cases, the repolarization of the rVAPw was incomplete, not reaching the baseline by the end of phase 3 (e.g., Figure 1C). Due to the spatial averaging effect of MCG, the mean values of all rVAPw repolarization duration parameters were longer than those of local eVMAP, though not significantly, although an opposite behaviour was observed in sessions n. 5 and 6. Only phase 0 duration of eVMAP was significantly shorter (Table 1).

**Figure 2:**
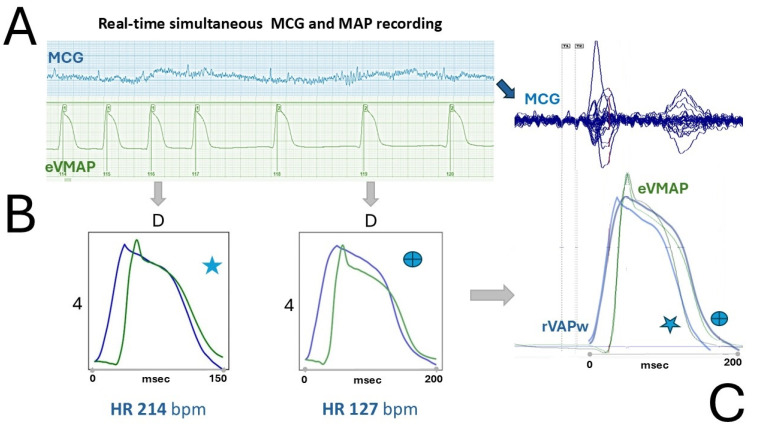
Example of ventricular repolarization adaptation to sudden heart rate decrease, as measured from magnetic rVAPw and simultaneous eVMAP. (**A**) Real-time recordings (left) and butterfly overlay of 36-channel averaged MCG signals; (**B**) Magnetic rVAPw (blue signal) and simultaneous eVMAP (green signal) obtained (grid position D4) with separate averaging of two epochs selected before (HR: 214 bpm) and after (HR: 127 bpm) sudden spontaneous heart rate variation (please note that the time scale at the same grid position D4 is different for the two epochs); (**C**) Overlay of rVAPw and eVMAP (both heart rates), after time scale normalization (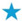 HR 214 bpm; 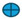 HR 127 bpm) clearly show the coherent shortening of both waveforms at the higher heart rate.

Although the need for averaging impeded the evaluation of rVAPw restitution during pacing-induced premature beats, heart rate-dependent shortening of the magnetic rVAPw was demonstrable in one session. A sudden, spontaneous drop of the heart rate from 214 bpm to 127 bpm (Figure 1A) lasted long enough to allow separate MCG averaging for two distinct heart rate epochs (Figure 2B).

At the lower heart rate, the prolongation of the averaged rVAPw duration-reconstructed from the MCG signal recorded at grid position D4-closely matched that of the simultaneously recorded eVMAP. This was confirmed by overlaying the waveforms averaged at the two heart rates, after time-scale normalization of the respective heart rate windows (Figure 2C).

### 3.3. Magnetic Action Potential Reconstruction at Different Ages

The mean values of depolarization and repolarization parameters, calculated from the reconstructed rVAPw of MCG sessions repeated in each animal at the ages of five, fourteen, and twenty-six months, are summarized in Table 2.

The data presented refer to rVAPw from two MCG channels (D3 and D4), arbitrarily selected for quantitative assessment among those showing the highest magnetic field signals. Only the duration of Phase 0 was significantly shorter at the age of five months compared to fourteen and twenty-six months.

**Figure 3:**
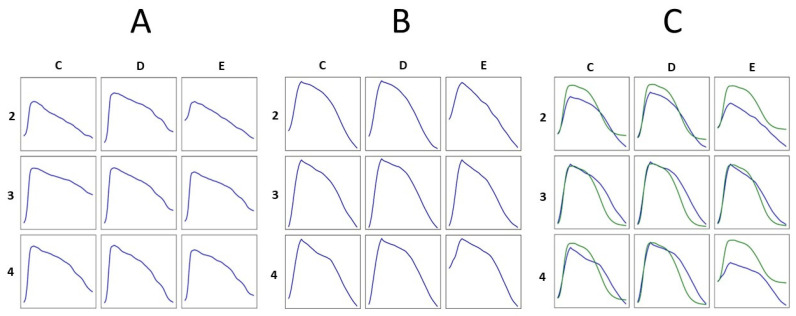
Examples of the morphological reproducibility of rVAPw calculated from MCG in the same animal at the ages of five (**A**) and twenty-six (**B**) months. (**C**) Comparison between rVAPw (blue waveform) and the simultaneously recorded epicardial ventricular MAP (green waveform) at the age of 26 months.

Graphic examples of rVAPw at different ages in the same animal are shown in Figure 3. Notably, the magnetically reconstructed action potential waveforms are reproducible, regardless of age or, consequently, weight.

### 3.4. Comparison Between Magnetically Reconstructed Action Potential in the Anaesthetized and Awake Conditions

The data for the magnetic action potential reconstructed from MCG recordings in the same animals, under both awake and anaesthetized conditions, are summarized in Table 3. The mean values of the rVAPw’s Phase 0 duration and repolarization durations, measured at d30%, d50%, and d80%, are presented. These values were calculated from two MCG channels (D3 and D4), which were arbitrarily selected for quantitative assessment based on their highest magnetic signal intensities.

**Figure 4:**
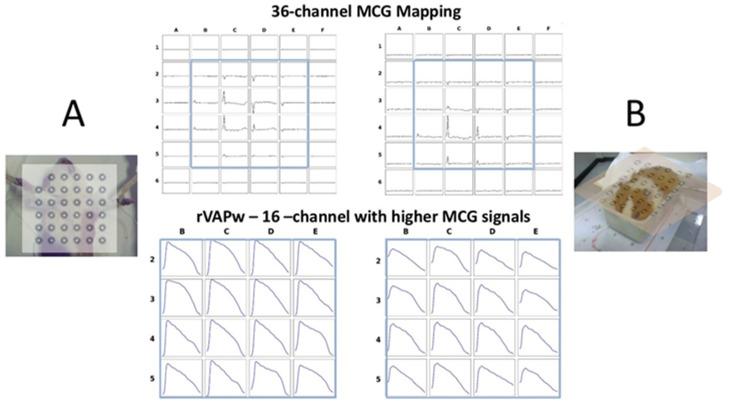
Magnetic action potential reconstruction and comparison between (**A**) anaesthetized and (**B**) awake conditions. The 36-channel sensor grid is overlayed on the animal in the supine and prone positions.

All repolarization parameters (except for d80% at position D4) were significantly shorter in the awake condition. This is likely not due to the different heart rates, which were slightly lower on average when the animals were awake, but rather to the varying spatial source sensitivity of MCG recordings from the anterior and posterior chest walls. However, we preferred to study the awake animal in its natural position to minimize the animal’s stress. Graphic examples of rVAPw from the same animal in both the anesthetized and awake conditions are shown in Figure 4.

### 3.5. Comparison Between Magnetic Action Potential Reconstruction in Males and Females

**Figure 5:**
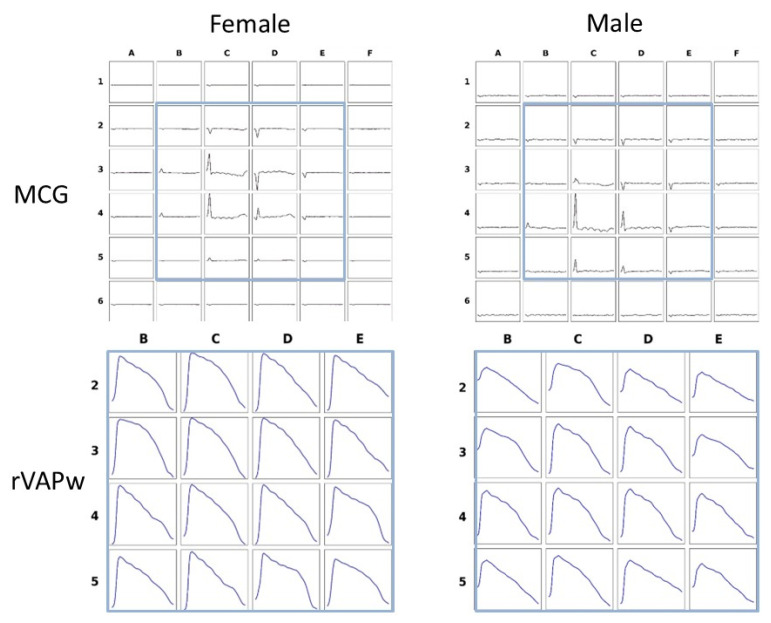
Examples of MCG signals mapped from the 36-point grid and the corresponding rVAPw are shown at the enlarged 4 × 4 selected grid sites with higher MCG signal intensity.

No gender-related differences were found in the rVAPw Phase 0 and repolarization intervals duration, calculated from the two MCG channels arbitrarily selected for quantitative assessment based on the highest magnetic signals. Graphic examples of rVAPw from one male and one female guinea pig are shown in Figure 5.

The data for the magnetic action potential, reconstructed from MCG recordings in eight male and ten female guinea pigs at the age of fourteen months, are summarized in Table 4.

## 4. Discussion

Preclinical regulatory studies of potential cardiotoxic and proarrhythmic side effects of new drugs, as well as investigations into genetically mediated arrhythmogenic mechanisms, are typically conducted using small experimental animal models [39,40,41,42,43,44]. However, non-invasive assessment of ventricular repolarization inhomogeneity in small experimental animals is generally limited to the 12-lead or vector ECG [45,46]. Although body surface potential mapping (BSPM) has been successfully reported [47,48], it is rarely used in practice due to being cumbersome and time-consuming.

An alternative to BSPM, contactless magnetocardiographic mapping, is feasible in intact rodents and even conscious guinea pigs [17,18]. MCG is sensitive to both intracellular and extracellular currents, thus providing electrophysiological information that ECG cannot offer, as ECG is only sensitive to extracellular currents [22,49,50,51,52].

The correlation between magnetic field imaging and optical mapping of cardiac action potentials was first explored in isolated rabbit hearts [53]. More recently, mathematical modeling of cardiac electrophysiology at the cellular level has enhanced the understanding of the relationship between the cardiac action potential, current density, and the associated magnetic field [54,55]. That research further supports the idea that recording cardiac magnetic fields can provide valuable physical information for detecting the mechanisms underlying cardiac arrhythmias at both experimental and clinical levels [54,55]. Notably, the findings from these modelling studies align with experimental results obtained by the same authors, which involved an optical mapping cross-species investigation of rabbit and guinea pig epicardial ventricular surfaces, as well as human endocardial tissue [56].

However, optical mapping is not feasible in intact experimental animals. While preliminary evidence suggests that ventricular action potential waveforms can be reconstructed from MCG recordings in human subjects [23,24], this study aimed to evaluate the feasibility of reconstructing ventricular action potential waveforms from MCG in small experimental rodents. This represents the first step toward developing a novel MCG-based device specifically designed for non-invasive, in vivo experimental mapping of ventricular action potential waveforms, suitable for large-scale preclinical safety studies in intact animals and, where possible, in conscious animals. To the best of our knowledge, no studies have been published on this topic to date.

The guinea pig was chosen for this study because its ventricular action potential closely resembles that of humans, and the guinea pig is docile enough to permit MCG mapping in the conscious state [57]. To avoid animal sacrifice, the preliminary validation of the magnetically reconstructed ventricular action potential waveform was conducted by comparing it with the ventricular monophasic action potential, recorded via a minimally invasive percutaneous approach using an amagnetic catheter placed in contact with the ventricular epicardium. This method allowed for full recovery and survival of the animal at the end of the procedure [25].

Overall, the results of this study confirm the feasibility of using uMCG mapping in guinea pigs weighing 250 g or more. After averaging 300 s of MCG recordings, the signal-to-noise (S/N) ratio of the averaged signals was sufficient to reconstruct the ventricular action potential from cardiac magnetic field mapping data. This was achieved despite conducting the procedure in a laboratory fully equipped for clinical interventional electrophysiology, using an unshielded mapping system designed for clinical measurements with a four-centimeter pitch between sensors. While this system provides adequate spatial resolution for the human heart, it is important to note that the area covered by the sensor array is comparable to the size of the entire guinea pig and is not optimized for the smaller size of the guinea pig’s heart.

On the other hand, our results demonstrated that, even under unfavourable experimental conditions—such as working in an unshielded hospital laboratory and using an MCG mapping device designed for clinical applications—the reconstruction of the ventricular action potential from uMCG was feasible in all the investigated animals. Furthermore, we observed suggestive individual morphological reproducibility of the rVAPw waveforms, particularly for those calculated from grid sites where MCG signals exhibited the highest intensity. The comparison of rVAPw obtained from MCG recordings at different ages in the same animal (Table 2) showed high morphological reproducibility of the ventricular repolarization course. The average duration of phase 0 of the rVAPw was significantly shorter at 5 months (a young adult) compared to 26 months (aged), while the average values for the repolarization phase remained virtually unchanged. A similar degree of reproducibility was observed when comparing rVAPw obtained from MCG recordings in the same animal at the same age under both awake and anesthetized conditions (Table 3), although MCG mapping was performed from the back (prone position) when the animal was awake, and from the anterior chest wall (supine position) under anesthesia (Figure 4). In line with a previous study of guinea pig magnetocardiograms [58], no gender-related differences in ventricular repolarization duration were found in adult guinea pigs, as measured from the rVAPw at 14 months of age (Table 4).

Preliminary validation of the magnetically reconstructed rVAPw, compared with the simultaneously recorded eVMAP waveform, demonstrates good morphological alignment. This alignment is further supported by the absence of significant differences in the average repolarization durations measured at the 30%, 50%, and 80% amplitude levels (Table 1).

Although the current need for signal averaging prevents assessment of rVAPw restitution during pacing-induced premature beats, heart rate-dependent shortening of the magnetic rVAPw was nevertheless observed. This was demonstrated by the concordant changes in rVAPw and eVMAP durations in one animal during a spontaneous heart rate drop from 214 bpm to 127 bpm (Figure 1A), which lasted long enough to permit separate MCG averaging for two distinct heart rate epochs (Figure 2B).

As expected, the average rVAPw duration was longer than that of the eVMAP, reflecting the spatial averaging inherent to surface MCG mapping compared to the higher local spatial sensitivity of MAP recordings. This represents one of the limitations of the present preliminary study.

In conclusion, the current level of MCG spatial resolution—at least with a mapping device designed for clinical applications—is still far from reaching the threshold required for rVAPw reconstruction accuracy sufficient for experimental electrophysiological studies, with diagnostic performance comparable to that achieved with optical mapping in in vitro or ex vivo experiments. However, the findings of this study may provide valuable insights for guiding the development of innovative instrumentation designed for contactless magnetic mapping of ventricular action potentials in small intact animal models, taking advantage of the limitations discussed in more detail in the following dedicated section.

## 5. Limitations and Lessons Learned

Despite demonstrating the feasibility of reconstructing the ventricular action potential waveform in guinea pigs, with acceptable individual reproducibility across recording sessions conducted over two-year follow-ups, our results highlight significant limitations. These findings indicate that further work is needed to translate the proposed method into a reliable, dedicated tool for the non-invasive electrophysiological study of intact experimental animal models, yielding data comparable to that obtained from optical mapping [2,53,56,59].

First, the MCG recordings conducted in our unshielded laboratories were influenced by environmental hospital noise, which required signal averaging to achieve a signal-to-noise ratio sufficient for the reliable analysis of the weak magnetic field from the guinea pigs’ hearts. As a result, the reconstruction of the action potential waveform was only possible from the averaged MCG signal, rather than on a beat-to-beat basis.

Although we were fortunate enough to quantify a sudden heart rate-dependent variation in the rVAPw repolarization restitution using differential averaging of selected MCG epochs and validated it through direct comparison with simultaneous eVMAP recordings (Figure 2), the need for averaging means that the quantitative estimation of the dynamics of ventricular repolarization restitution is currently not feasible. Consequently, this limits the ability to detect transient spontaneous or drug-induced arrhythmogenic events that may favour spatially discordant alternans and re-entry [54,55,56]. This limitation needs to be addressed and could potentially be overcome by implementing real-time, novel AI-assisted denoising algorithms combined with small-size electromagnetic shielding [60].

Second, as mentioned earlier, while it is mathematically possible to extract the cardiac action potential waveform from uMCG signals, it is important to note that the MCG signal is derived from the spatial averaging of the current density generated by an undefined volume of underlying anisotropic cardiac tissue. This spatial averaging effect in MCG may explain the partial distortion of the rVAPw or the incomplete agreement observed between the morphology of rVAPw and that of eVMAP (e.g., Figure 1C). Furthermore, the accuracy of the rVAPw may be partly influenced by the method used to approximate electric current values, which can be affected by the distance between measurement sites. In this study, the large distance between sites, with a four-centimeter pitch of sensors in our MCG system, may contribute to this influence. Notably, since the relationship between the current (“I”) and the vertical component of the magnetic field (Bz) is highly localized, the calculation of current sources located at the center of the sensors is not significantly affected by the lower stability observed near the boundary regions. Increasing the spatial density and resolution of sensors would reduce the MCG averaging effect over the magnetic fields generated by multiple ventricular myocytes of varying natures (e.g., Purkinje fibers, M-cells, etc.) during data acquisition. This would provide a more realistic approximation of the transmembrane action potentials at least at the tissue level. This could be achieved by combining rVAPw reconstruction from MCG recorded with novel microfabricated millimeter-scale sensor arrays [61,62], high-resolution 3D magnetic source imaging [63], and integration with cardiac MRI [64]. Given that the propagation of cardiac currents is three-dimensional, a higher sensor density capable of simultaneously recording all three vector components of the cardiac magnetic field is essential [65].

Third, novel hardware should be complemented by more advanced computational modeling, including more accurate simulations of ionic currents [55,66,67], and a better understanding of how local action potentials from different regions of the heart correlate with the magnetic fields measured by MCG. This would enhance the accuracy of electric current (“I”) calculations and, consequently, enable more localized action potential reconstruction and differentiation.

Finally, while setting the resistance to a constant value of 1 in the formulas may not significantly affect the conceptual outcome [23], the computations should ideally account for average conductivity values, at least as averages across different types of cardiac myocytes and layers of cardiac tissue. The implementation of this adjustment would substantially increase the computational complexity of the model, requiring more powerful computational resources.

## 6. Conclusions

In this study, five-minute uMCG recordings were sufficient to non-invasively reconstruct the rVAPw in intact—and even awake—guinea pigs weighing 250 g or more, without causing harm to the animals. However, the atypical and partially incomplete repolarization patterns observed at several mapping sites suggest that the spatial resolution of the clinical 36-channel system used for MCG mapping is limited. Enhancing the 3D sensor density and spatial resolution is a minimal requirement for accurate, high-resolution measurement of cardiac magnetic field dynamics, and for distinguishing the underlying cardiac sources and electrophysiological events. Therefore, selecting the most appropriate sensor technology is essential to optimize the 3D spatial accuracy required for precise AP reconstruction [61,62].

In addition, advanced modelling and signal processing techniques, including deep learning algorithms [68], could enable multimodal integration of rVAPw mapping with MCG-based 3D source localization [69] and magnetoionographic parameters sensitive to transmembrane calcium and potassium currents, as well as subcellular Ca^2+^ transients [70].

Future advancements in magnetic sensor technologies, combined with AI-driven integration of biophysical models of cardiac electrophysiology and magnetism, and techniques such as magnetic induction tomography [71] and magnetoionography [70] may ultimately support fully non-invasive, high-resolution, real-time MCG-based assessment and imaging of cardiac electrophysiology in intact and awake experimental animals. This would establish MCG as a uniquely powerful non-invasive tool for experimental electrophysiological imaging, offering data quality comparable to that of invasive optical mapping [24]. The same method has been recently successfully applied to reconstruct ventricular action potential waveforms from clinical magnetocardiographic mapping in patients with different electrophysiological abnormalities and as well as in healthy volunteers [72].

## Figures and Tables

**Table 1:** Individual values of phase 0 and repolarization intervals duration.

				eVMAP				rVAPw			
MCG Session	AGE	Weight	HR	Phase 0	d30%	d50%	d80%	Phase 0	d30%	d50%	d80%
	months	grams	bpm	msec	msec	msec	msec	msec	msec	msec	msec
1	29	650	172	19	163	177	195	18	182	214	218
2	29	650	157	14	100	185	208	16	114	169	218
3	29	650	204	17	72	94	116	22	56	86	140
4	29	650	200	19	66	90	122	29	88	110	142
5	28	615	213	15	61	95	128	21	48	79	119
6	28	615	193	16	89	116	134	19	57	87	126
7	26	422	142	18	91	111	142	30	108	136	175
8	28	600	198	17	53	119	167	29	86	121	151
mean	28.3	606.5	184.9	16.9 ◊	86.9	123.4	151.5	23.0 ◊	92.4	125.3	161.1
SD	1.0	77.3	25.1	1.8	34.7	37.2	34.7	5.6	43.7	46.8	38.9

eVMAP: epicardial ventricular monophasic action potential; rVAPw: ventricular action potential reconstructed waveform from simultaneous MCG mapping. ◊: *p* < 0.05.

**Table 2:** Comparison of Phase 0 and repolarization duration, calculated from rVAPw, at different ages.

	Age Sessions (months)						
	5		14		26		
rVAPw	Mean	SD	Mean	SD	Mean	SD	*p*
Phase 0	19	3	20	2	23	4	<0.01
D3 d30%	53	16	51	19	50	18	ns
D4 d30%	52	15	51	20	49	17	ns
D3 d50%	82	19	79	23	74	21	ns
D4 d50%	81	19	80	24	72	20	ns
D3 d80%	113	19	111	19	107	19	ns
D4 d80%	112	20	112	21	108	20	ns
HR	256.3	46.7	250.7	44.7	253.1	49.0	ns

HR: heart rate (bpm); other parameters in msec; rVAPw: magnetically reconstructed ventricular action potential waveform; ns: not significant.

**Table 3:** Comparison of Phase 0 and repolarization duration, calculated from rVAPw, in anaesthetized and awake conditions.

	Anaesthetized		Awake		
rVAPw	Mean	SD	Mean	SD	*p*
Phase 0	20	3	20	4	ns
D3 d30%	57	19	41	8	<0.01
D4 d30%	55	19	44	13	0.01
D3 d50%	86	21	67	15	<0.01
D4 d50%	84	22	69	18	<0.01
D3 d80%	116	19	102	15	<0.01
D4 d80%	114	21	106	19	ns
HR	257.7	46.9	245.5	43.8	ns

HR: heart rate (bpm); other parameters in msec; rVAPw: magnetically reconstructed ventricular action potential waveform; ns: not significant.

**Table 4:** Comparison of Phase 0 and repolarization duration, calculated from rVAPw, in males and females.

	Males		Females		
rVAPw	Mean	SD	Mean	SD	*p*
Phase 0	20	2	19	2	ns
D3 d30%	48	11	53	24	ns
D4 d30%	51	14	52	25	ns
D3 d50%	77	15	81	28	ns
D4 d50%	79	19	80	29	ns
D3 d80%	112	14	111	24	ns
D4 d80%	114	17	111	24	ns
HR	248.3	44.7	252.8	46.2	ns

HR: heart rate (bpm); other parameters in msec; rVAPw: magnetically reconstructed ventricular action potential waveform; ns: not significant.

## Data Availability

The unique datasets presented in this article are not readily available because the data are part of other ongoing research studies. Moreover, for institutional regulation, proprietary research data can be shared with external institutions only within the framework of officialized research cooperation agreements.

## Abbreviations

The following abbreviations are used in this manuscript:

MCG	Magnetocardiography
uMCG	Unshielded MCG
rVAPw	reconstructed Ventricular Action Potential waveform
GPs	Guinea pigs
eVMAP	epicardial Ventricular Monophasic Action Potential
AP	Action Potential
fT	femtotesla
kHz	Kilohertz
CAM	Current Arrow Map
MFD	Magnetic Field Distribution
3D	Three-dimensional
EMD	Effective Magnetic Dipole
msec	Milliseconds
HR	Heart Rate
